# Age-associated differences in XBB.1.5 trivalent booster vaccine-induced adaptive responses revealed by single-cell RNA sequencing

**DOI:** 10.1080/22221751.2026.2627067

**Published:** 2026-02-03

**Authors:** Shixing Chen, Tao Liu, Jing Chen, Shengxia Yin, Jinqiu Ran, Wen Zhang, Wanying Zhang, Juan Zhang, Chen Li, Xun Wang, Pengfei Wang, Chao Wu, Fan Yang, Yuxin Chen

**Affiliations:** a Department of Infectious Diseases, Nanjing Drum Tower Hospital, Affiliated Hospital of Medical School, Nanjing University, Nanjing, People’s Republic of China; b Institue of Biomedicine and Biotechnology, Shenzhen Institutes of Advanced Technology, Chinese Academy of Sciences; c Department of Laboratory Medicine, Joint Institute of Nanjing Drum Tower Hospital for Life and Health, College of Life Science, Nanjing Normal University, Nanjing, People’s Republic of China; d Department of Laboratory Medicine, Nanjing Drum Tower Hospital Clinical College of Nanjing Medical University, Nanjing, People’s Republic of China; e Shanghai Pudong Hospital, Fudan University Pudong Medical Center, State Key Laboratory of Genetics and Development of Complex Phenotypes, MOE Engineering Research Center of Gene Technology, School of Life Sciences, Shanghai Institute of Infectious Disease and Biosecurity, Shanghai Key Laboratory of Oncology Target Discovery and Antibody Drug Development, Fudan University, Shanghai, People’s Republic of China; f Department of Laboratory Medicine, Nanjing Drum Tower Hospital, Affiliated Hospital of Medical School, Nanjing University, Nanjing, People’s Republic of China

**Keywords:** SARS-CoV-2, XBB.1.5 booster vaccination, age-associated immune responses, B cell clonal dynamics, single cell RNA sequencing

## Abstract

Older adults remain highly vulnerable to severe SARS-CoV-2 outcomes despite multiple vaccinations, yet age-associated differences in immune responses to updated COVID-19 booster vaccines remain incompletely characterized. Here, we administered an XBB.1.5 trivalent recombinant protein booster (WSK-V102C) to 22 individuals (<38 years) and 20 individuals (≥73 years), all of whom had previously received 2–3 doses of inactivated COVID-19 vaccines. Neutralizing antibody responses against multiple SARS-CoV-2 variants were quantified and compared between age groups. Meanwhile, single-cell RNA sequencing was also performed on peripheral blood mononuclear cells (PBMCs) collected at baseline and 28 days post-vaccination to profile age-associated immune features following boosting. Following booster immunization, both age groups achieved significantly elevated antibody titres against all tested strains. Nevertheless, the magnitude of antibody fold increase was consistently lower in elderly individuals than in younger adults. Single-cell analyses revealed age-associated differences in post-vaccination immune organization. In elderly individuals, B-cell state transitions were characterized by transcriptional signatures consistent with memory B cell-to-plasmablast differentiation, whereas younger individuals predominantly exhibited transitions from naïve B cells. CD4+ T cells from elderly individuals displayed altered transcriptional trajectories and reduced T-cell receptor diversity relative to younger adults. In contrast, younger individuals showed coordinated B- and T-cell-associated transcriptional programmes, including enrichment of transcription factors such as KLF7, CEBPB, CEBPD, and MAFB. Collectively, our study describes age-associated differences in immune coordination and cellular response patterns following XBB.1.5 booster vaccination. Further longitudinal and functional studies will be required to clarify the mechanistic basis and clinical implications of these observations.

## GRAPHICAL ABSTRACT

**Figure:**
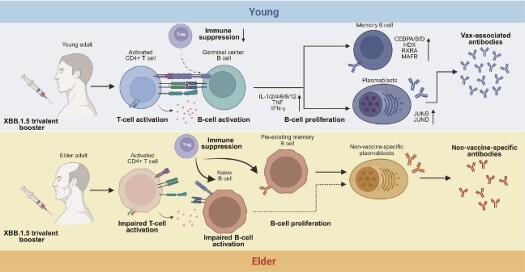

## Introduction

Older adults remain disproportionately vulnerable to severe illness and mortality from COVID-19, even after global vaccination campaigns [1,2]. Age-associated changes in the immune system have been linked to altered vaccine responsiveness, which may contribute to a sustained burden of breakthrough infections and adverse clinical outcomes in this population. Despite widespread administration of primary and booster COVID-19 vaccines, accumulating evidence suggests age-related differences in humoral and cellular immune responses, although the underlying immunological features associated with these differences remain incompletely understood [3,4].

The emergence and rapid global dissemination of the SARS-CoV-2 XBB lineage at the end of 2022 further underscored these challenges. By late 2023, XBB co-circulated with Omicron BA.2.86, eventually giving way to JN.1 by mid-2024. Nevertheless, recent data indicate that XBB-induced immune responses retain cross-protective potential against JN.1 and related subvariants [5,6]. In this context, effective booster strategies are urgently needed-particularly for the elderly-to counteract ongoing immune escape and reinfection risk [7]. The World Health Organization currently recommends that high-risk populations, including older adults, receive a COVID-19 booster every 6–12 months to maintain protective immunity [8]. However, limited data exist regarding the cellular and molecular features of booster responses in individuals aged ≥70, especially in the context of variant-specific vaccines.

Age-related declines in immune competency have been associated with the reduced effectiveness of multiple vaccines [9]. This attenuation arises not only from physiological changes associated with aging but also from intrinsic alterations within the immune system of older adults [10,11]. Aging is commonly accompanied by a chronic, low-grade inflammation state, often referred to as inflammaging [12]. Chronic inflammation accelerates the senescence of immune cells, leading to impaired immune function and a reduced capacity to clear senescent cells and inflammatory mediators [13]. Vaccine efficacy depends on the generation of protective antibodies, a process orchestrated by interactions between B cells and CD4+ T cells [3]. Both B and T lymphocytes originate from hematopoietic stem cells in the bone marrow, whose regenerative capacity declines with advancing age [14]. The thymus is essential for T cell development but undergoes progressive involution and degeneration with age, resulting in a marked reduction in T-lymphocyte numbers and restricted antigen receptor diversity in older adults [15,16]. T cell aging is further characterized by the downregulation of co-stimulatory molecules such as CD27 and CD28, as well as a propensity for naive CD4+ T cells in older individuals to differentiate into short-lived effector T cells rather than memory T cells upon activation [17]. Insufficient pools of antigen-specific candidate T cell clones may contribute to the slower immune responses to inactivated SARS-CoV-2 vaccines observed in older adults [4]. Aging likewise impairs B cell function, leading to reductions in total B cell numbers and subset diversity, which in turn decreases plasma cell output, narrows the B cell receptor repertoire, and weakens vaccine-elicited antibody responses [18,19]. Moreover, age-associated B cells have been shown to diminish vaccine responsiveness by attenuating affinity maturation during germinal centre reactions [20]. In addition, multiple studies have reported an age-dependent decline in vaccine-induced neutralizing antibodies for both mRNA and inactivated vaccines [21–23].

Although these studies have yielded critical insights, they were largely conducted in the context of ancestral or early Omicron strains, using either inactivated or mRNA vaccine platforms. As immune-evading variants such as XBB.1.5 emerge, and variant-adapted vaccines are deployed globally, the mechanisms underlying age-related heterogeneity in immune response to these updated formulations remain poorly defined. Particularly lacking are single-cell transcriptomic data comparing young and elderly responses to XBB.1.5-adapted vaccines, which could uncover age-specific shifts in immune subset composition, activation programmes, and cell–cell communication networks.

WestVac BioPharma’s Coviccine® Trivalent XBB Vaccine (WSK-V102C) is the first vaccine globally approved for emergency use against the XBB.1.5 variant. Clinical studies have demonstrated that this vaccine elicits robust neutralizing antibody responses across multiple SARS-CoV-2 variants, with high seroconversion rates and favourable immunogenicity profiles compared with earlier bivalent Omicron vaccines [24]. Yet, the extent to which these responses differ across age groups – especially at the level of antigen-specific T and B cell function – has not been well characterized.

To address these gaps, we conducted a comprehensive single-cell RNA sequencing (scRNA-seq) analysis of peripheral immune responses to WSK-V102C. In this study, we recruited 22 young and 20 elderly adults, all of whom had previously received two or three doses of inactivated vaccines, and administered a booster with the WSK-V102C vaccine. By leveraging single-cell transcriptomic, B cell receptor (BCR) and T cell receptor (TCR) sequencing, we systematically dissected the transcriptional profiles, clonal dynamics, and intercellular communication patterns of peripheral immune cells in each age group. This analysis allowed us to characterize age-associated differences in post-vaccination immune features, including transcriptional states, clonal architecture, and inferred cell-cell communication patterns. This study provides a descriptive framework for understanding age-related immune organization following booster vaccination and highlights directions for future mechanistic and longitudinal investigation.

## Materials and methods

### Ethics statement

This study was conducted in accordance with the principles outlined in the Declaration of Helsinki. Ethical approval was granted by the Research Ethics Committee of Nanjing Drum Tower Hospital (Approval No. 2023-609-02). Written informed consent was obtained from all participants for both sample collection and subsequent analyses.

### Participant enrollment and clinical sample collection

A total of 42 healthy participants (including 21 males and 21 females) were enrolled at Nanjing Drum Tower Hospital from December 2023 to January 2024 (Tables S1 and S2). To assess age-associated immune responses, the cohort comprised 22 younger individuals (12 males and 10 females) aged 23–38 years and 20 elderly individuals (9 males and 11 females) aged 73–86 years. All participants had no known history of systemic diseases, including, but not limited to, hepatitis B or C, HIV, diabetes, kidney or liver diseases, malignant tumours, or autoimmune diseases. All participants received a single dose of WestVac BioPharma’s Coviccine® Trivalent XBB Vaccine (WSK-V102C), which is composed of recombinant receptor binding domains (RBDs) derived from the XBB.1.5, BA.5, and Delta SARS-CoV-2 variants, adjuvanted with a squalene-based oil-in-water emulsion [25]. Prior to enrolment, all participants had received either two or three doses of inactivated COVID-19 vaccines, with the most recent dose administered at least 12 months before booster vaccination. All participants had a documented history of laboratory-confirmed SARS-CoV-2 infection between December 2022 and January 2023. No participant reported symptomatic reinfection or tested positive for SARS-CoV-2 within the six months preceding the booster vaccination. For the scRNA-seq analyses, an external healthy control cohort was included for baseline comparison. This cohort comprised age- and sex-matched individuals (n = 12) with no reported history of COVID-19 vaccination or prior SARS-CoV-2 infection. The control datasets were retrieved from publicly available Gene Expression Omnibus (GEO) databases (accession numbers: GSE157007 and GSE213516) and were used as a reference for baseline immune features (Table S3).

Peripheral blood samples were collected immediately prior to vaccination (Day 0) from all participants. Follow-up samples were obtained at Day 28 post-vaccination from 40 participants; samples were unavailable from two young participants (y10 and y12). All samples were collected within this same enrolment period. To ensure technical consistency and minimize batch effects, sample processing from peripheral blood mononuclear cell (PBMC) isolation to library preparation was performed using standardized protocols, identical reagent lots, and consistent instrument settings. Samples were randomized across experimental batches to prior to processing.

### Construction and production of variant pseudoviruses

Plasmids encoding the spike proteins of various Omicron sub-lineages and the wild-type (WT) SARS-CoV-2 spike were constructed and used for pseudovirus production. These spike plasmids were transfected into HEK293 T cells using polyethylenimine (PEI) (Polysciences, lnc.). The cells were incubated overnight at 37°C with 5% CO_2_. The next day, the transfected cells were infected with vesicular stomatitis virus G protein (VSV-G) pseudotyped ΔG-luciferase virus (G*ΔG-luciferase; Kerafast) at a multiplicity of infection (MOI) of 5. After 4 h of incubation, the cells were washed three times with 1× Dulbecco’s phosphate-buffered saline (DPBS) to remove residual input virus. Culture supernatants containing pseudoviruses were harvested the following day and clarified by centrifugation at 3,000 × g for 10 min. To neutralize any remaining VSV-G contamination, I1 hybridoma supernatant (anti-VSV-G; ATCC CRL-2700) was added to each viral preparation and incubated at 37°C for 1 h. Pseudovirus titres were then quantified, and viral stocks were aliquoted and stored at −80°C until use.

### Pseudovirus neutralization assays

Pseudovirus-based neutralization assays were conducted to evaluate the neutralizing capacity of serum samples by measuring the reduction in luciferase expression. Heat-inactivated sera were initially diluted at 1:40 and serially diluted 5-fold across 96-well plates in triplicate. Each well received 1,000 TCID₅₀ of pseudovirus, which was incubated with the serial serum dilutions at 37°C for 60 min. During the incubation period, Vero-E6 cells were harvested via trypsinization, resuspended in fresh culture medium, and added to each well at a density of 2 × 10⁴ cells per well. The plates were then incubated at 37°C for 24 h. Luciferase activity was quantified using the Luciferase Assay System (Beyotime), and neutralization was assessed based on the reduction in luminescence. The 50% inhibitory concentration (IC_50_) was defined as the serum dilution at which relative light units (RLUs) were reduced by 50% compared to virus control wells (virus + cells), after subtraction of background RLUs from cell-only control wells. IC_50_ values were calculated using nonlinear regression analysis in GraphPad Prism (v10.1.1). To compare neutralizing antibody titres before and after vaccination between the elderly and young cohorts, a two-tailed Wilcoxon matched-pairs signed-rank test performed in R (v4.3.1) was used. A *p*-value < 0.05 was considered statistically significant.

### Single-cell RNA library preparation and sequencing

Cell suspensions were barcoded using 10x Chromium Single Cell platform using Chromium Single Cell 5’ Library, Chromium Single Cell 3’ Library and Gel Bead Kit (10x Genomics), following the manufacturer’s instructions. Briefly, fluorescence-activated cell sorting (FACS) was performed to isolate target cells, which were then washed three times with 0.04% BSA in PBS. The cells were resuspended to a final concentration of 700–1200 cells/μL with ≥85% viability, as assessed by the Countess™ II Automated Cell Counter (Thermo Fisher Scientific). Single cells were encapsulated into droplets using the Chromium Controller to achieve targeted cell recovery. Reverse transcription was carried out within the droplets, after which emulsions were broken and barcoded cDNA was purified using Dynabeads (Thermo Fisher Scientific). The purified cDNA underwent PCR amplification. Amplified cDNA was split into two workflows, including 5’ gene expression library construction and T cell receptor (TCR)/B cell receptor (BCR) enrichment. For gene expression libraries, 50 ng of amplified cDNA was fragmented, end-repaired, and subjected to double-sided size selection using SPRIselect beads (Beckman Coulter). Libraries were then sequenced on an Illumina NovaSeq platform to generate 150 bp paired-end reads. For TCR/BCR libraries, TCR and BCR transcripts were selectively amplified through 10 cycles of PCR using kit-provided primers targeting the V(D)J regions. The resulting libraries were size-selected using SPRI beads and sequenced on an Illumina NovaSeq 6000 system with 150 bp paired-end reads. All raw sequencing data were processed using the Cell Ranger pipeline (10x Genomics) with default settings for gene expression quantification and immune repertoire analysis.

### Single-cell RNA-seq data processing and statistical analysis

Quality control and preprocessing of single-cell RNA-seq data were performed using the Seurat package (v4.3.0.1) [26]. Cells were retained if they expressed more than 200 and fewer than 6,000 genes and had a mitochondrial unique molecular identifier (UMI) rate below 10%. Mitochondrial and ribosomal genes were excluded from the expression matrix prior to downstream analysis. Normalization and scaling were carried out using Seurat’s standard workflow, including regression on UMI counts and mitochondrial gene content to mitigate technical variation. Highly variable genes (n = 3,000) were identified using the *FindVariableFeatures* function. Principal component analysis (PCA) was then performed on the variable genes using the *RunPCA* function.

To account for potential technical variation and sample-to-sample heterogeneity, data integration was performed using the Harmony package (v0.1.1) implemented through *RunHarmony* function. Harmony was applied during the integration step to mitigate batch effects associated with sample processing and library preparation across different experimental runs. Furthermore, to reduce the influence of demographic and vaccination-related covariates on downstream comparisons between age groups, Harmony integration was conducted while adjusting for sex and the number of prior vaccine doses, as previously described [27]. This approach allowed alignment of shared biological variation across samples while minimizing confounding effects attributable to these covariates in subsequent analyses.

Graph-based clustering was subsequently performed using the top 18 principal components with a resolution parameter set to 0.6, yielding unsupervised cell clusters. Marker genes for each cluster were identified using the *FindAllMarkers* function based on the Wilcoxon rank-sum test, using the following criteria: (1) log fold change (logFC) > 0.25; (2) *p*-value < 0.05; (3) minimum percentage of expressing cells (min.pct) > 0.25. For refined cell type identification, clusters presumed to represent the same cell type were grouped and subjected to re-clustering using Uniform Manifold Approximation and Projection (UMAP), followed by graph-based clustering and additional marker gene analysis.

### Single-cell immune profiling data analysis

BCR analysis was initiated using the output files generated by the Cell Ranger V(D)J pipeline (10x Genomics). V(D)J gene segments were annotated by aligning sequences against the International ImMunoGeneTics (IMGT) reference database using IgBlast (v1.21.0) [28]. Sequences identified as nonproductive – those containing stop codons or frameshifts – were excluded from subsequent analyses.

To identify clonal relationships, B cells were grouped into clonal clusters based on shared V(D)J ancestry, inferred through somatic hypermutation patterns. Initially, sequences were categorized by common IGHV and IGHJ gene annotations. Clonal clusters were then defined using a normalized Levenshtein distance threshold of 0.2 within the CDR3 region, employing single-linkage hierarchical clustering [29]. All BCR analyses were performed in R (v4.3.1).

Vaccine-correlated B cell clones were classified based on their presence and expansion patterns post-vaccination. Clones were excluded from this analysis if they were already present before vaccination or showed limited expansion (i.e. cell count < 3). A “vaccine-correlated clone” was defined as any BCR clonotype that met one or more of the following criteria: (1) exclusively detected post-vaccination in the same individual with a cell count ≥ 3, thereby qualifying as a large clone; (2) demonstrated increased clonal expansion post-vaccination within the same individual, relative to pre-vaccination level; (3) detected across at least 3 individuals in post-vaccination samples, suggesting convergent clonal responses to the vaccine.

TCR repertoire profiling was conducted using the Cell Ranger V(D)J pipeline. The pipeline assembled raw reads into V(D)J contigs, identified cell barcodes from targeted cells, and annotated sequences with V, D, and J gene segments, while precisely locating the CDR3 region. Only high-confidence contigs, as determined by Cell Ranger, were retained for downstream analysis. Cells containing ambiguous or incomplete TCR chains (e.g. missing β chains or ambiguous α/β pairings) were excluded. In cases where multiple α and/or β chains were detected in a single cell, the chain with the highest nUMI value was selected to ensure unique clonotype assignment. No evidence of cross-sample contamination was observed, confirmed by analysis of barcode and contig sequence overlaps. T cells were grouped into clonotypes based on identical CDR3 sequences in both α and β chains, allowing for assessment of T cell clonal expansion and diversity across samples.

To infer cellular developmental trajectories using pseudo-time analysis, we converted the integrated Seurat object to h5ad format using the SeuratDisk package (version 0.0.0.9020), enabling compatibility with the Scanpy pipeline. The data were merged with a corresponding loom file [30]. A neighbourhood graph was constructed based on the top 20 principal components using *scanpy.pp.neighbors*. We then applied *scanpy.tl.diffmap* to build the diffusion map, and the first two diffusion components were used for visualization [31].

To systematically investigate intercellular signalling, we performed cell–cell communication analysis using the CellChat framework, which leverages a curated database of ligand–receptor interactions and cofactors [32]. We utilized the CellChatDB.human database to predict predominant signalling inputs and outputs across identified single-cell clusters. To assess transcription factor regulatory activity, we implemented the pySCENIC workflow (version 0.9.5), incorporating the 20,000-motif database from RcisTarget and regulatory inference through GRNBoost [33]. The resulting loom file was imported into the SCENIC R package (version 1.3.1) for downstream visualization and interpretation of transcriptional regulatory networks.

Differentially expressed genes (DEGs) were identified using standard parameters in the muscat package (version 1.13.1) [34]. Functional enrichment was assessed via both Gene Set Enrichment Analysis (GSEA) and Gene Set Variation Analysis (GSVA), implemented using the clusterProfiler package (version 4.8.3), which enables robust statistical analysis and visualization of gene and pathway-level profiles [35]. To explore lineage transitions among immune cell populations, we followed an adapted developmental dynamics framework [36]. Transitions between B cell clusters and CD4+ T cell clusters were quantified using the pTrans index derived from STARTRAC analysis [37].

## Results

### Age-associated differences in neutralizing antibody responses following XBB.1.5 booster vaccination

To compare the humoral immune responses between young and elderly individuals after administration of the WSK-V102C vaccine, serum neutralizing antibody titres against the ancestral D614G strain and five SARS-CoV-2 variants (B.1.617.2, BA.2.87.1, BA.5, XBB.1.5, and JN.1) were measured using a pseudovirus neutralization assay. Prior to vaccination, all participants exhibited baseline neutralizing activity against the tested strains. Following booster immunization, both groups achieved significantly elevated antibody titres against all variants examined. Specifically, elderly individuals showed 7.00-, 9.55-, 13.38-, 11.87-, 9.93-, and 9.68-fold increases in geometric mean titres (GMTs) against D614G, B.1.617.2, BA.2.87.1, BA.5, XBB.1.5, and JN.1, respectively (Figure S1(A); all *p* < 0.001). In contrast, young individuals demonstrated more robust responses, with GMTs increasing by 7.07-, 7.88-, 16.15-, 17.88-, 15.01-, and 23.46-fold against the corresponding variants, respectively (Figure S1(B); all *p* < 0.001). Notably, direct comparisons of post-vaccination fold-changes revealed consistently higher increases in young individuals than in elderly individuals across six variants (Figure S1(C)). The fold increase in young individuals was most pronounced against XBB.1.5 (3.8-fold, *p* = *0.001*), followed by BA.5 (3.4-fold, *p* = *0.006*), BA.2.87.1 (3.1-fold, *p* < *0.001*), JN.1 (2.3-fold, *p* = *0.006*), D614G (2.1-fold, *p* < *0.001*), and B.1.617.2 (1.6-fold, *p* = *0.012*). Importantly, despite substantial overlap in absolute post-vaccination neutralizing antibody titres between age groups, the consistently smaller fold increases observed in elderly individuals, highlighting an age-associated difference in the relative magnitude of antibody responses following booster vaccination.

### Dynamic single-cell transcriptional atlas of peripheral immune cells in young and elderly cohorts before and after vaccination

**Figure 1.**
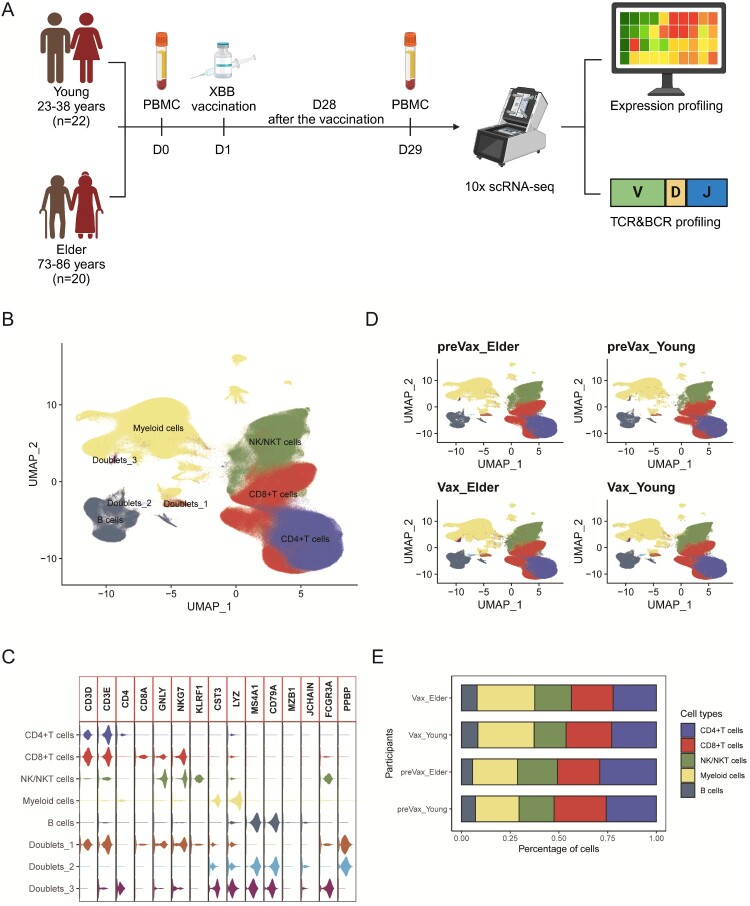
Study design and patient cohort. **A**, Schematic overview of the study design. Peripheral blood samples were collected from 42 donors (21 males and 21 females), and single-cell RNA sequencing (scRNA-seq) was performed on PBMCs. The resulting datasets were used for transcriptomic profiling as well as TCR and BCR repertoire analyses. **B**, UMAP projection of 882,324 single cells derived from 80 PBMC samples, identifying eight major immune cell populations. Each dot represents a single cell, coloured by annotated cell type. **C**, Violin plots showing the expression of selected canonical marker genes across the eight immune cell populations. **D**, UMAP visualization of immune cell clusters coloured by experimental group. **E**, Box plots showing the proportional abundance of five major cell types by participant, excluding doublets.

To investigate age-related differences in immune responses, we performed scRNA-seq (10x Genomics) on PBMCs between young and elder groups (Figure 1(A)). PBMCs samples were collected prior to vaccination and 28 days post-vaccination, respectively. In total, 92 PBMC samples were included in the analysis, comprising 80 prospectively collected samples from our cohort and 12 healthy controls sourced from public datasets. Specifically, these included young pre-vaccination (preVax_Young; n = 20), young post-vaccination (Vax_Young; n = 20), elderly pre-vaccination (preVax_Elder; n = 20), and elderly post-vaccination (Vax_Elder; n = 20) from our study, supplemented by 6 healthy young controls (HC_Young) and 6 healthy elderly controls (HC_Elder). Additionally, T cell receptor (TCR) and B cell receptor (BCR) sequencing was performed for every individual. After quality control and filtering, we retained 882,324 high-quality single-cell transcriptomes.

Unsupervised graph-based clustering using Uniform Manifold Approximation and Projection (UMAP) identified five major immune cell populations and three doublet clusters, classified based on canonical marker gene expression (Figure 1(B)). The major clusters included CD4+ T cells (CD3D + CD3E + CD4+), CD8+ T cells (CD3D + CD3E + CD8A+), NK/NKT cells (GNLY + NKG7 + KLRD1+), B cells (MS4A1 + CD79A+), and myeloid cells (CST3 + LYZ+) (Figure 1(C)). Notably, the overall distribution of these immune cell types remained consistent across all four groups, with no significant compositional differences observed (Figure 1(D and E)).

### Impaired maturation and antigen-specific differentiation observed in the elderly following booster immunization

A total of five B cell clusters were identified, including B_Naive, B_Memory_Unswitched, B_Memory_Class_Switched, B_Cycling, and Plasmablasts (Figure 2(A and C)). No significant differences were observed in the overall proportion of B cells across the four groups (Figure 2(B and D)). To further examine the transcriptional responses of B cells following booster vaccine, we performed differential gene expression analysis on each B cell subset before and after boosting (Figure S2(A–D)). Gene set enrichment analysis (GSEA) revealed that, after boosting, the B_Naive and B_Memory_Class_Switched cells from young individuals exhibited significant enrichment of gene sets associated with lymphocyte activation, proliferation, differentiation, inflammatory response, adaptive immune response, and cytokine response, compared to their counterparts in the elderly individuals (Figures 2(E, F) and S2(E)). Additional enrichment of V(D)J recombination and cell adhesion pathways in these clusters suggests specific activation and immunoglobulin gene assembly processes in young individuals. These processes may also promote CD4+ T cell activation via MHC-II-mediated antigen presentation.

**← Figure 2.**
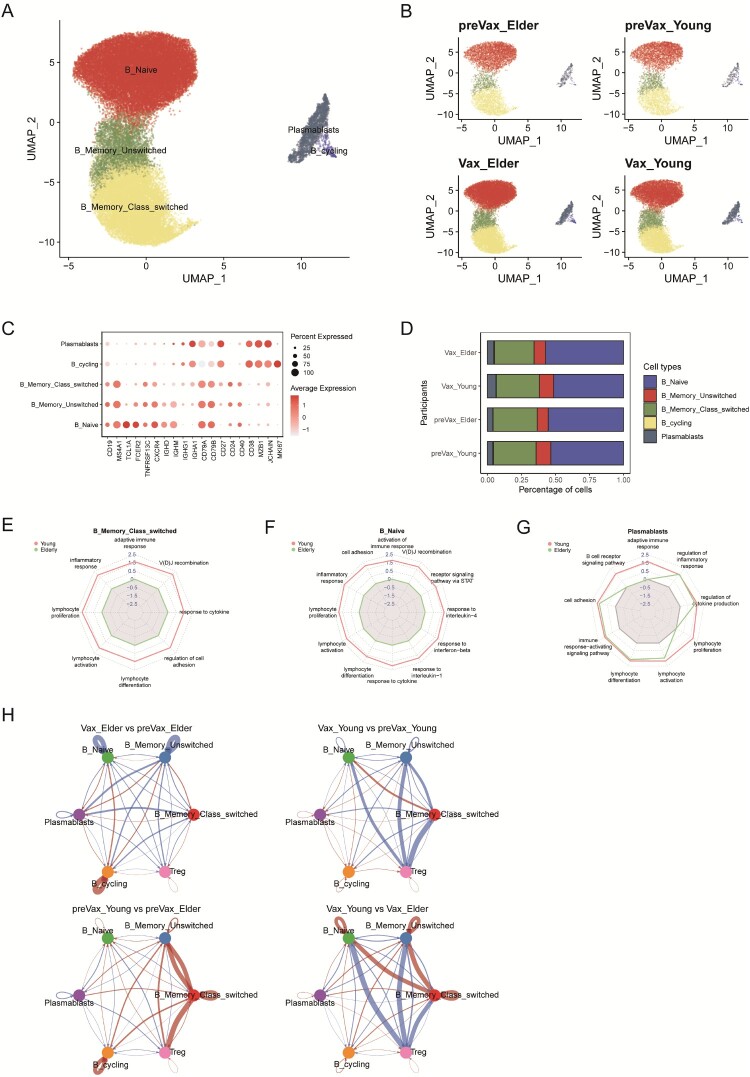
Immunological features and comparative analysis of the B cell populations. **A**, UMAP projection of B cell subsets identified from PBMCs. **B**, UMAP plots of B cell subsets coloured by experimental group. **C**, Dot plot showing the expression of representative marker genes across B cell subsets. **D**, Box plots depicting the proportional abundance of the B cell subsets per participant. **E–G**, Gene set enrichment analysis (GSEA) of B cells in young and elderly individuals. Radar plots display normalized enrichment scores (NES) for selected functional gene sets within individual B cell subsets. NES > 0 indicates enrichment in the leading group, whereas NES < 0 indicates enrichment in the trailing group. **H**, Comparison of inferred cell-cell interaction strength of B cells and regulatory T cells (Tregs) across conditions: Vax_Elder vs. preVax_Elder (top left), preVax_Young and preVax_Elder (bottom left), Vax_Young and preVax_Young (top right), and Vax_Young as well as Vax_Elder (bottom right). Blue lines indicate decreased interactions and red line indicates increased interaction; line thickness reflects relative interaction strength.

In contrast, B cells from elderly individuals lacked significant enrichment in pathways related to B cell signalling, activation, and immune response, with the exception of the Plasmablasts, which showed moderate enrichment in vaccine-responsive pathways (Figures 2(G) and S2(F)). Although Plasmablasts in both age groups displayed enrichment for lymphocyte activation, differentiation, inflammatory response, and cell adhesion, these effects were consistently stronger in young individuals (Figure 2(F)). To assess whether these age-associated differences reflected responses to the current booster vaccination rather than pre-existing immunological differences, we compared pre-vaccination samples (preVax_Young and preVax_Elder) with age-matched unvaccinated healthy controls (HC_Young and HC_Elder) using GSEA with the identical gene sets (Figure S2(G)). At baseline, B_Naive cells from young individuals exhibited enrichment primarily in cell adhesion-related pathways and displayed relatively weaker adaptive immune response signature compared with elderly individuals. Similarly, B_Memory_Class_Switched cells from young individuals exhibited enrichment restricted to pathways involved in cell adhesion. Notably, plasmablasts from both age groups showed no significant enrichment in vaccine-responsive pathways at baseline. Together, these observation suggest that the baseline transcriptional states of B cell subsets in the pre-vaccination cohort were broadly comparable to those observed in unvaccinated individuals, supporting the interpretation that the transcriptional changes observed after boosting predominantly reflect responses associated with the XBB.1.5-adapted vaccine. Consistent with this, B_Naive and B_Memory_Class_Switched cells from elderly individuals exhibited weaker activation-related transcriptional responses following WSK-V102C vaccination compared with those from young individuals.

Comparative enrichment analyses further revealed age-related transcriptomic differences prior to vaccination. B_Naive and B_Memory_Class_Switched cells in pre-vaccination elderly individuals were enriched in pathways related to inflammatory response, cytokine signalling, adaptive immunity, cell adhesion, and cellular respiration, compared to the preVax_Young group (Figure S2(H)). However, this apparent enrichment likely reflects B cell senescence and a chronic inflammatory milieu associated with aging rather than enhanced immune functionality. Supporting this interpretation, post-vaccination samples from young individuals (Vax_Young) showed markedly higher enrichment of similar gene sets than those from elderly individuals (Vax_Elder), particularly within B_Naive and B_Memory_Class_Switched cells (Figure S2(I)). Furthermore, B_Naive cells in Vax_Young individuals showed upregulation of pathways critical for B–T cell cooperation and differentiation, including signalling pathways involving interleukins, TNF, IFN-γ, and the JAK-STAT axis.

To explore the regulatory landscape of B cell responses, we investigated cell-cell communication between B cell clusters and regulatory T cells (Tregs). The preVax_Young group exhibited the highest number and strength of interactions, which markedly declined after vaccination (Vax_Young) (Figure S2(J)). In contrast, the interaction network in elderly individuals remained relatively unchanged before and after vaccination. Notably, pre-vaccination interactions between B cells and Tregs were significantly more extensive in young individuals compared to elderly individuals, whereas the post-vaccination profile was reversed (Figure 2(H)). This shift was largely due to a sharp reduction in interactions between Tregs and B_Naive/B_Memory cells in young individuals after vaccination, suggesting that Tregs in young individuals may exert more dynamic immune regulatory control, while this regulatory function is diminished with aging.

Collectively, these findings highlight age-associated differences in the transcriptional response of B cell subsets to WSK-V102C vaccination. Specifically, limited activation of B_Naive and B_Memory cells, along with weakened regulatory suppression by Tregs, may partially contribute to the diminished vaccine responsiveness observed in elderly individuals. In contrast, Plasmablasts, although activated in both age groups, do not appear to account for the reduced immunogenicity in the elderly at the transcriptomic level.

### Memory-skewed B cell activation and impaired germinal center-like maturation in the elderly

**Figure 3.**
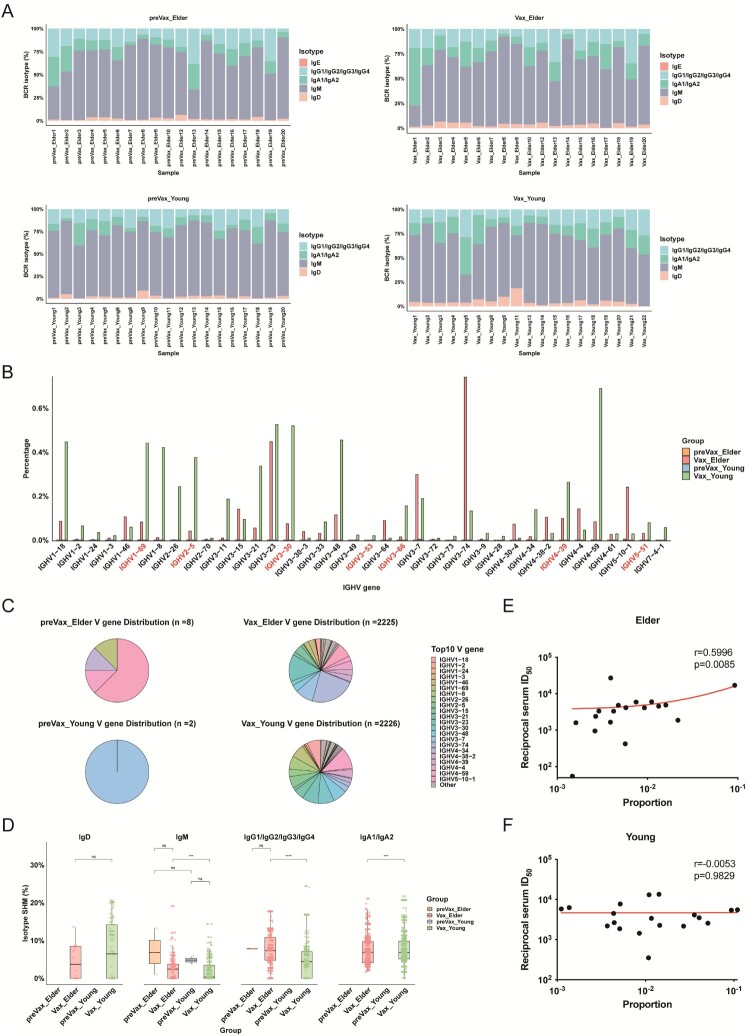
B cell clonal architecture and BCR V(D)J features following XBB.1.5 trivalent booster vaccination. **A**, Stacked bar chart showing the distribution of IgD, IgM, IgA, IgG, and IgE isotypes across samples in preVax_Elder (top left), preVax_Young (bottom left), Vax_Elder group (top right), and Vax_Young (bottom right) groups. **B**, IGHV gene usage in Vax-correlated B cells across age groups. IGHV genes previously reported to be associated with SARS-CoV-2 responses are highlighted in red. **C**, Pie charts showing the distribution of IGHV gene usage among Vax-correlated B cells, with the top 10 IGHV genes labelled for each group. **D**, Comparison of somatic hypermutation (SHM) levels across B cell isotypes in Vax-correlated B cells between groups. Group differences were assessed using Fisher’s exact test (ns, no significant difference; *** *p* < 0.005; **** *p* < 0.001). **E–F**, Spearman correlation analyses between the proportion of Vax-correlated B cells and serum neutralizing antibody titres (ID50) against the XBB.1.5 variant in young and elderly individuals, respectively.

To investigate the clonal architecture and V(D)J gene usage of B cells in response to WSK-V102C vaccination, we reconstructed BCR sequences from single-cell BCR sequencing data. Analysis of immunoglobulin isotype distribution revealed no significant differences in the proportions of IgD, IgM, IgA, IgG, or IgE between young and elderly individuals, either before or after vaccination (Figure 3(A)). To identify vaccine-responsive B cell populations, we defined “Vax-correlated clones” as specific BCR clones that either expanded significantly following vaccination or were shared across multiple individuals. The corresponding B cells were designated as “Vax-correlated B cells,” serving as a proxy for antigen-driven selection and expansion triggered by vaccination.

Following vaccination, both young and elderly groups demonstrated significant expansion of B cell clones compared to their pre-vaccination baselines. Analysis of Vax-correlated B cells revealed preferential usage of specific immunoglobulin heavy chain variable (IGHV) genes, with young individuals exhibiting a significantly higher frequency of SARS-CoV-2-related IGHV genes than their elderly counterparts. Notably enriched genes included IGHV3-53/3-66, IGHV5-51/4-39, and IGHV1-69/3-30/2-5 (Figure 3(B and C)). Among these, IGHV3-53 and IGHV3-66 are well-characterized public clonotypes commonly involved in the generation of Class 1 neutralizing antibodies targeting the receptor-binding motif (RBM) of the SARS-CoV-2 spike protein [38]. In contrast, IGHV5-51 and IGHV4-39 have been frequently associated with immune responses against SARS-CoV-2 Omicron subvariants including XBB.1.5 and JN.1. Additionally, IGHV1-69, IGHV3-30, and IGHV2-5 have been preferentially utilized in Omicron-specific monoclonal antibodies (mAbs) that recognize distinct epitope regions, such as E1/E2.1, F1.1, and F3, supporting their relevance in variant-specific immunity [39]. In contrast, clonal expansion in elderly individuals was characterized by the predominant expansion of a limited number of dominant clones, most notably those utilizing IGHV3-23 and IGHV3-74.

Somatic hypermutation (SHM) levels were significantly elevated in Vax-correlated B cells following WSK-V102C vaccination in both young and elderly individuals, indicating activated geminal centre engagement and affinity maturation (Figure 3(D)). Interestingly, SHM levels of IgM, IgA, and IgG B cells were consistently higher in elderly individuals compared to young individuals [40]. This phenomenon likely reflects the cumulative accumulation of mutations in long-lived memory B cells in the elderly. Alternatively, it supports the notion that WSK-V102C vaccination preferentially reactivates memory B cells in the elderly individuals, rather than inducing *de novo* responses from naive B cells, as observed in the young individuals.

Further analysis revealed a significantly positive correlation between the proportion of Vax-correlated B cells and neutralizing antibody titres (ID50) against XBB.1.5 variants in elderly individuals (*p* = 0.0085, R = 0.5996; Figure 3(E and F)). In contrast, no such correlation was observed in young cohort. This age-dependent difference suggests that the relationship between B cell clonal expansion and antibody output differs between the two groups. In young individuals, neutralizing antibody titres did not scale linearly with the abundance of vaccination-correlated B cells, indicating a more complex contribution of multiple B cell subsets to the overall antibody response. In elderly individuals, by contrast, antibody titres showed a stronger association with the expansion of vaccination-correlated B cells, consistent with a response dominated by a limited number of expanded clones. Notably, clonal expansion in elderly individuals was largely driven by a small set of dominant B cell clones, particularly those utilizing IGHV3-23 and IGHV3-74. Together, these observations highlight age-associated differences in the organization of B cell responses following WSK-V102C vaccination, with elderly individuals exhibiting a more direct correspondence between clonal expansion and antibody production.

### Impaired CD4+ T cell differentiation with elevated Treg-mediated suppression in the elderly

**Figure 4.**
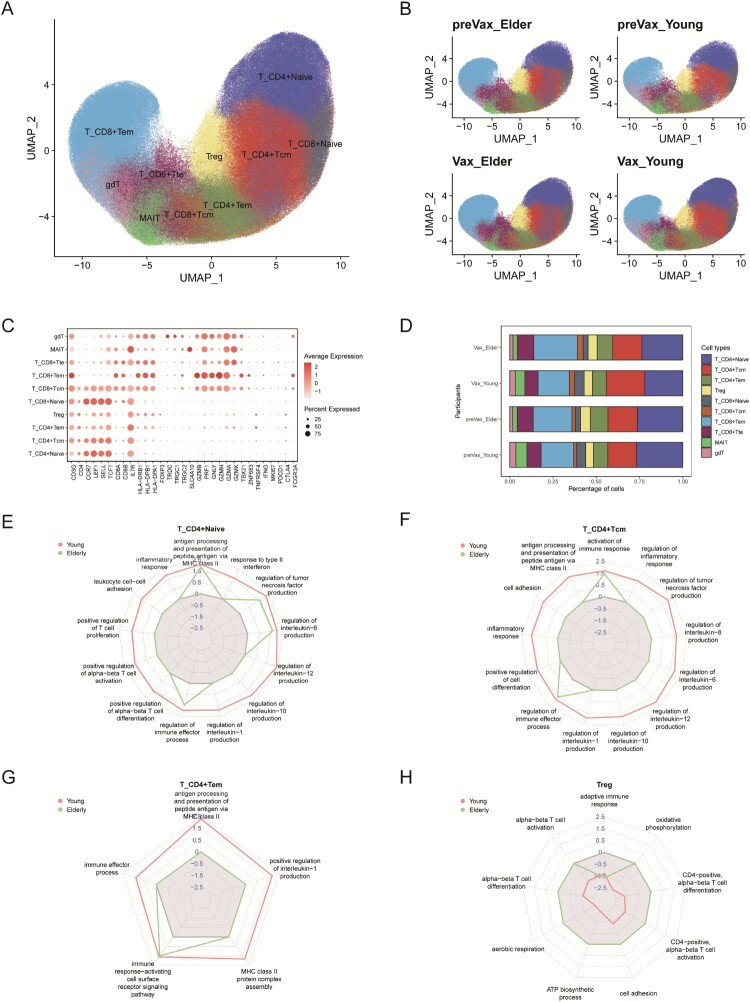
Immunological features and comparative analysis of the CD4+ T cell types. **A**, UMAP projection of the CD4+ T cell subsets. **B**, UMAP plots of CD4+ T cell subsets coloured by experimental group. **C**, Dot plot showing the expression of representative marker genes across CD4+ T cell subsets. **D**, Box plots depicting the proportional abundance of CD4+ T cell subsets per participant. **E–H**, GSEA analysis of CD4+ T cells between young and elderly individuals. Radar plots display normalized enrichment scores (NES) for selected functional gene sets within individual CD4+ T cell subsets.

Single-cell transcriptomic analysis identified 10 major T cell clusters, including T_CD4 + Naive, T_CD4 + Central Memory (Tcm), T_CD4 + Effector Memory (Tem), Tregs, T_CD8 + Naive, T_CD8 + Tcm, T_CD8 + Tem, T_CD8 + Terminal Effector (Tte), MAIT, and γδT cells (Figure 4(A and C)). Among these, the relative proportions of CD4+ T cell subsets remained stable across age groups and vaccination status (Figure 4(B and D)). To evaluate functional responses, we performed differential gene expression (DEG) and gene set enrichment analysis (GSEA) on CD4+ T cell subsets (Figure S3(A–D)). In young individuals, both T_CD4 + Naive and T_CD4 + Tcm cells exhibited significant enrichment in gene sets related to T cell activation, differentiation, inflammatory response, MHC class II antigen presentation, and cytokine production compared to elderly individuals’ post-vaccination (Figure 4(E and F)). T_CD4 + Tem cells in young individuals also showed strong enrichment for pathways associated with immune effector functions, protein complex assembly, and antigen presentation, suggesting enhanced effector capacity (Figure 4(G)).

In elderly individuals, CD4+ T cells also responded to vaccination, with enriched pathways including adaptive immunity, dendritic cell antigen presentation, and cell surface receptor-mediated immune signalling, albeit at lower intensity compared to young individuals (Figure S3(E)). Notably, Treg-mediated immunosuppressive signatures were significantly elevated in the elderly, whereas Treg activity was reduced in young individuals’ post-vaccination (Figure 4(H)), indicating diminished regulatory restraint and potentially more robust immune activation in the young cohort. In response to WSK-V102C vaccination, T_CD4 + Naive and T_CD4 + Tcm cells from young individuals exhibited marked transcriptional activation, with the upregulation of genes involved in interleukin signalling (e.g. IL-1, IL-6, IL-8, IL-10, IL-12), MHC class II-mediated antigen presentation, and cell adhesion (Figure S3(F)). These changes suggest enhanced interactions between CD4+ T cells and antigen-presenting cells, such as B cells and dendritic cells. Collectively, these findings support that WSK-V102C vaccination promotes CD4+ T cell differentiation toward antigen-presentation–associated effector subsets, such as T follicular helper (Tfh) cells, thereby facilitating B cell proliferation, class switching, and IgG antibody production [41,42]. To evaluate whether these age-associated differences reflected responses to the current booster vaccination rather than pre-existing immune states, we compared pre-vaccination samples with age-matched unvaccinated healthy controls using the same GSEA framework (Figure S3(G)). At baseline, T_CD4 + Tcm cells from young individuals showed significant enrichment primarily in antigen processing and presentation via MHC class II, while exhibiting weaker enrichment in pathways related to positive regulation of cell differentiation compared with elderly individuals. No other CD4+ T cell subsets, including T_CD4 + Naive, T_CD4 + Tem, or Treg cells, showed significant enrichment of vaccine-responsive pathways prior to boosting. Together, these observations suggest that the baseline transcriptional states of CD4+ T cell subsets in the study cohort were broadly comparable to those observed in unvaccinated healthy individuals. Accordingly, the enhanced transcriptional activation observed in CD4+ T cell subsets from young individuals after boosting is more likely associated with post-vaccination immune responses rather than pre-existing immunological differences.

In contrast, prior to vaccination, elderly individuals exhibited higher enrichment of MHC-II, cell adhesion, and adaptive immune response within the T_CD4 + Tcm subset compared to young individuals (Figure S3(H)), potentially reflecting chronic immune activation or a memory skewed phenotype. However, compared to their elderly counterparts, vaccinated young individuals displayed greater enrichment of interleukin-regulated immune signalling, and adhesion pathways within T_CD4 + Naive and T_CD4 + Tcm cell subsets (Figure S3(I)), highlighting age-related differences in CD4+ T cell responsiveness.

### CD4+ T cells in the elderly displayed constrained clonal expansion and impaired differentiation into effector and helper subsets

**Figure 5.**
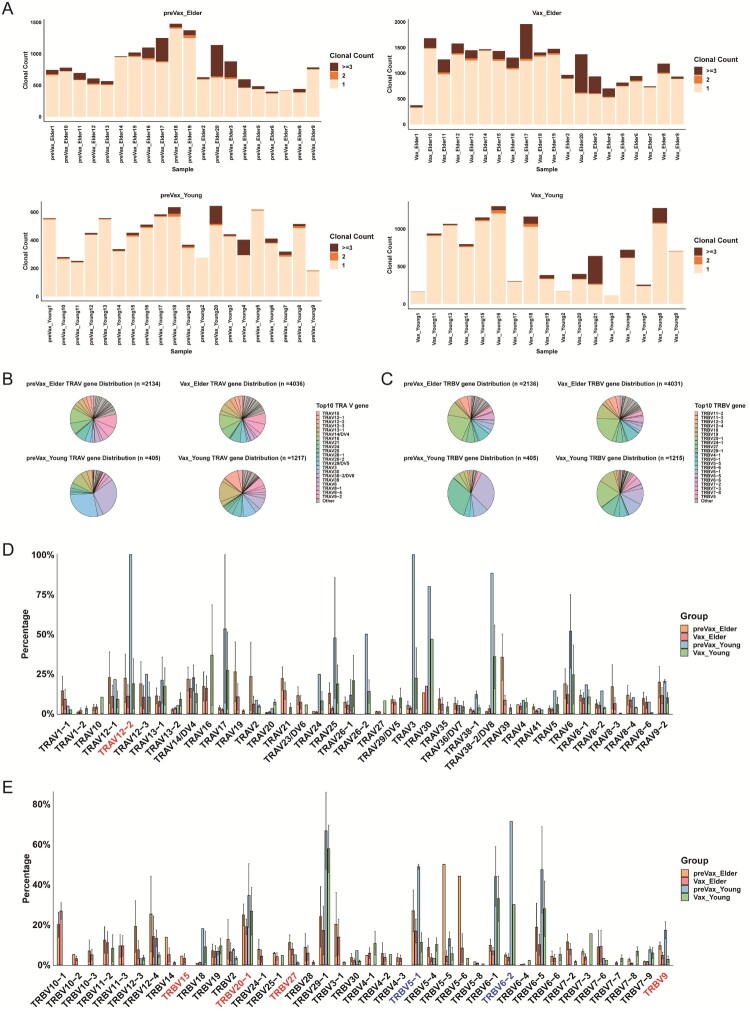
CD4+ T cell clonal diversity and TCR V(D)J features after booster vaccination. **A**, Stacked bar plots showing CD4+ T cell clonal diversity across samples in preVax_Elder (top left), preVax_Young (bottom left), Vax_Elder (top right), and Vax_Young (bottom right) groups. **B–C**, Pie charts showing the distribution of TRAV (B) and TRBV (right) gene usage among Vax-correlated CD4+ T cells, with the top 10 genes labelled for each group. **D–E**, Comparative analysis of TRAV (D) and TRBV (E) gene usage in Vax-correlated CD4+ T cells across groups. Genes with relatively higher and lower usage compared with reference SARS-CoV-2-associated repertoires are highlighted in red and blue, respectively.

To further dissect CD4+ T cell activation and clonal dynamics in response to WSK-V102C vaccination, we reconstructed TCR sequences from scTCR-seq data. Firstly, clonal expansion of T cells was evident in both young and elderly individuals prior to vaccination (Figure 5(A)), consistent with prior antigen exposure or the presence of cross-reactive memory T cells. Following vaccination, Vax-correlated T cell clones were detected in both age groups. Despite comparable overall expansion, TRAV and TRBV gene usage patterns remained largely unchanged in elderly individuals, suggesting that vaccination primarily reactivated pre-existing memory T cell clones rather than generating new vaccine-specific repertoires. In contrast, young individuals exhibited a dynamic remodelling of the TCR repertoire, characterized by a downregulation of TRAV and TRBV genes with highly used prior to vaccination (Figure 5(B and C)). This included both genes with previously high frequencies in COVID-19 patients (e.g. TRAV12-2, TRBV9, and TRBV15) and those with lower frequencies (e.g. TRBV5-1 and TRBV6-2), all of which showed a consistent decline in usage post-vaccination in both age group (Figure 5(D and E)) [43,44]. This shift may reflect the emergence of XBB-specific TCR repertoires, potentially involving upregulation of clonotypes such as TRAV10**,** TRAV26-1, and TRBV19, which may target novel epitopes introduced by XBB.1.5 variant. Alternatively, this pattern could result from natural contraction of activated T cells by day 28 post-vaccination, as part of the resolution phase of the immune response. Taken together, these findings suggest that TCR repertoires in elderly individuals remain relatively static, dominated by memory recall responses, while young individuals exhibit more flexible and adaptive clonal remodelling, potentially generating *de novo* XBB-specific T cell responses.

### Vaccine-induced B cell responses in the elderly exhibited reduced clonal specificity and impaired memory B–CD4+ T cell interactions

To examine age-related differences in B cell responses to vaccination, we performed gene set variation analysis (GSVA) on B cell functional signatures (Figure S4(A)). In young individuals, B_Naive, B_Memory_Unswitched, and B_Memory_Class_Switched cells showed significant enrichment in gene sets associated with adaptive immune responses and acute inflammation, indicating robust vaccine-induced activation. In contrast, B cells from elderly individuals lacked such enrichment post vaccination, despite showing higher baseline activation signatures before vaccination, which likely reflects chronic low level of inflammation commonly observed in aging [45]. To further explore immune aging, we generated an age-associated B cell (ABC) gene signature, which revealed increased ABC enrichment post-vaccination in the elderly but not in the young [46]. These findings suggests that the vaccination may further accelerate B cell senescence and immune exhaustion in the elderly individuals, potentially impairing vaccine efficacy.

**Figure 6.**
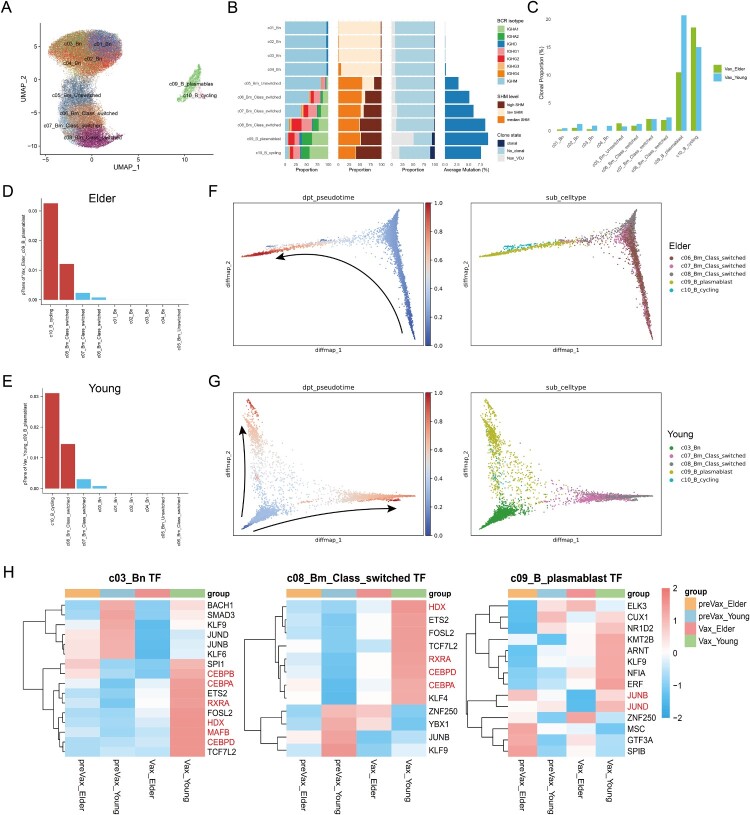
Cellular communication and transcriptional characteristics of B cell subsets. **A**, UMAP projection of B cell subsets. **B**, Distribution of IgH isotypes, SHM levels, proportion of Vax-correlated B cells, and median SHM rate across B cell subset. **C**, Proportion of clonal cells within B cell subsets stratified by age group. **D–E,** Pairwise transition index (pTrans) of c09 B cells with other B cell subsets in elderly (D) and young (E) individuals, respectively; the top two transitions are highlighted. **F,** Pseudo-time differentiation trajectory for selected B cell subsets from elderly individuals. The black arrow indicates the dominant trajectory observed. **G,** Pseudo-time differentiation trajectory inferred for selected B cell subsets from young individuals. Black arrows indicate two major trajectories observed. **H,** Heatmap showing area under the curve (AUC) scores of transcription factors for selected B cell subsets estimated using SCENIC.

Unsupervised clustering identified 10 B cell subclusters, including naive B cells (Bn; c01–c04), unswitched memory B cells (Bm_Unswitched; c05), class-switched memory B cells (Bm_Class_Switched; c06–c08), plasmablasts (c09), and cycling B cells (c10) (Figure 6(A)). Isotype analysis revealed clear class-switching patterns. Specifically, Bn subsets predominantly expressed IgM and IgD, Bm_Class_Switched cells and plasmablasts progressively expressed greater levels of IgA and IgG, consistent with class-switch recombination (CSR) and SHM (Figure 6(B)) [47,48].

Although overall clonal expansion was comparable between age groups, plasmablasts in young individuals contained a significantly higher proportion of Vax-correlated clones (Figure 6(C)). This suggests that young individuals more effectively generate antigen-specific plasmablasts, contributing to a stronger antibody response. In contrast, elderly individuals produced fewer vaccine-specific B cell clones, despite similar total expansion, highlighting qualitative defects in their humoral responses (Figures 3 and 6(C)).

Gene expression profiling further distinguished functional roles of Bm subsets. The c06 cluster was enriched for ABC markers, MHC-II, and antigen presentation genes, indicating a potential role in CD4+ T cell activation. The c08 cluster, in contrast, expressed high levels of activation markers, transcription factors, inflammatory cytokines, and chemokine receptors, reflecting an activated B cell phenotype (Figure S4(B)). Subclusters c05 and c07 showed limited functional gene expression.

CellChat-based interaction analyses revealed that subclusters c03, c05, c06, and c08 interacted extensively with CD4+ T cells in both age groups (Figure S4(C)). Notably, c08 subsets expressed the highest number of costimulatory ligand-receptor pairs, including CD86–CD28, CD99–CD99, ITGAM_ITGB2–CD40LG/ICAM2, facilitating T cell activation (Figure S4(E and F)) [49,50]. Moreover, memory B cells from young individuals exhibited more ligand–receptor pair interactions with CD4+ T cells than those from the elderly (Figures S4(D)), suggesting a greater capacity to support T cell activation, differentiation, and proliferation, and immune coordination.

Subsequently, STARTRAC transition index and pseudo-time analysis was used for the analysis of B cell maturation trajectories. In elderly individuals, c06-c08 subsets predominantly transitioned into plasmablasts, likely secreting non-XBB-specific antibodies (Figures 6(D–F) and S4(G–I)) [36,37]. In contrast, young individuals followed two distinct differentiation trajectories. One trajectory originated from c03_Bn subset, progressing through a germinal centre-like activation pathway and culminating in the generation of plasmablasts. The other trajectory led to the formation of memory B cell subsets, suggesting the establishment of long-term immunological memory (Figure 6(G)). These findings reflect a more functionally diverse and antigen-specific B cell maturation in the young population, involving both effector differentiation and memory cell development.

Transcription factor (TF) analysis revealed age-related dependent regulatory programmes. In young individuals, CEBPA, CEBPB, CEBPD, HDX, RXRA, and MAFB were highly expressed in B subsets excluding plasmablasts, suggesting their involvement in promoting cell growth, differentiation, and anti-senescence related transcriptional programmes (Figure S4(J)). In contrast, JUNB and JUND were selectively upregulated in plasmablasts after vaccination, indicating a potential role in driving terminal B cell differentiation and antibody-secreting cell formation. While these TFs are known mediators of inflammation and aging, their specific roles in variant-directed immunity remained to be elucidated [51]. Our data showed that young individuals mount a more effective B cell response characterized by robust activation of naive B cells and antigen-specific maturation, whereas elderly individuals predominantly rely on memory B cells that tend to differentiate into non-specific plasmablasts.

### The XBB.1.5 trivalent booster preferentially engages c07 and c09 CD4+ T cell subsets with age-divergent responses

**Figure 7.**
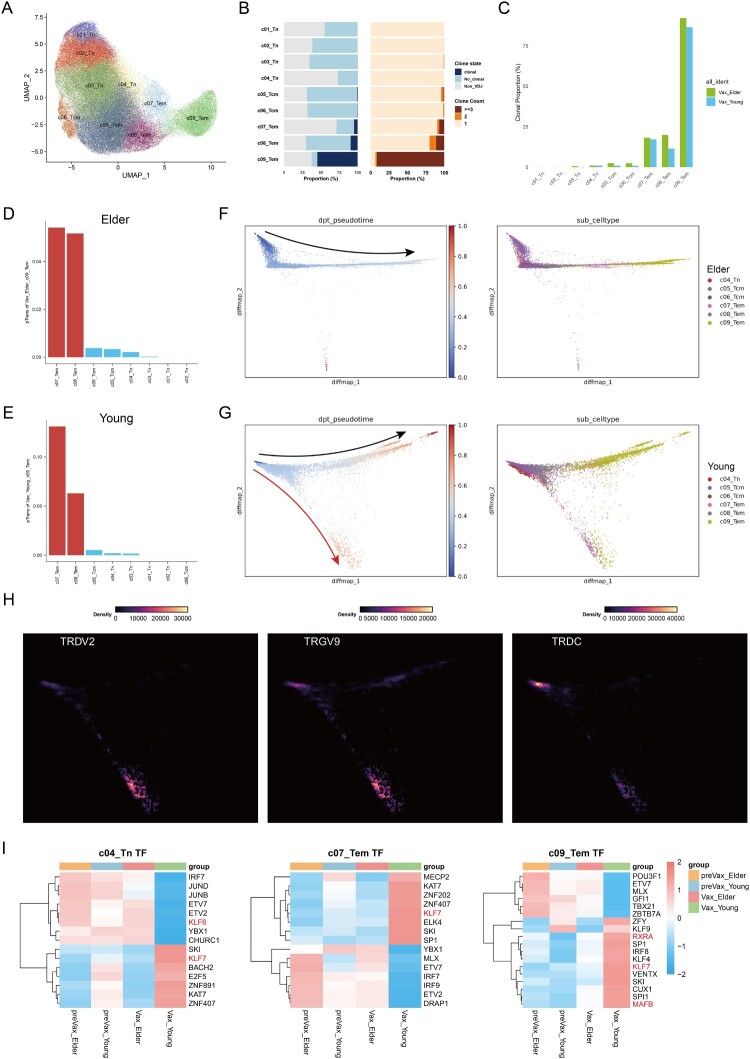
Cellular communication and transcriptional characteristics of CD4+ T cell subsets. **A**, UMAP projection lot of CD4+ T cells subsets. **B**, Statistical analysis of the proportions of Vax-correlated CD4+ T cells across subsets. **C**, Proportion of clonal cells within CD4+ T cell subsets stratified by age group. **D–E,** Pairwise transition index (pTrans) of c09 CD4+ T cells with other CD4+ T cell subsets from elderly (D) and young (E) individuals, with the top two subsets highlighted. **F–G,** Pseudo-time differentiation trajectories inferred for CD4+ T cell subsets from elderly (F) and young (G) individuals. Black arrows indicate shared trajectories, while the red arrow indicates the trajectory uniquely observed in young individuals. **H,** Pseudo-time visualization highlighting TCR genes with elevated expression along the young-specific trajectory. **I,** Heatmap showing the area under the curve (AUC) scores of transcription factors for selected CD4+ T cell subsets estimated using SCENIC.

To investigate the heterogeneity of CD4+ T cell response to vaccination, we performed unsupervised clustering, identifying nine CD4+ T cell subclusters, including naive T cells (Tn; c01–c04), central memory T cells (Tcm; c05–c06), and effector memory T cells (Tem; c07–c09) (Figure 7(A)). Among these, the c09 subset exhibited the highest enrichment of Vax-correlated CD4+ T cells post-vaccination **(**Figure 7(B)**)**. Gene expression profiling of the memory subsets revealed that the c07 and c09 subsets expressed elevated levels of age-associated T cell (ATC) markers, MHC-II, and antigen processing genes, while c07 additionally expressed high levels of inflammatory cytokines and chemokine receptors (Figure S5(A)) [4,52].

Clonal expansion analysis showed no significant differences in the distribution of Vax-correlated CD4+ T cell clones between age groups, although the overall proportion of clonal CD4+ T cells was slightly higher in elderly individuals (Figure 7(C)). These findings suggest that while both young and elderly individuals generated clonal CD4+ T cells post-vaccination, the c07 and c09 subsets likely represent the major vaccine-responsive populations.

Cellular communication analysis using CellChat revealed robust interactions between CD4+ T subsets and B cells in both age groups (Figure S5(B and C)). Specifically, c07 and c09 Tm subsets exhibited the most extensive ligand-receptor interactions with B cells, including KLRB1-CLEC2B and ITGAM_ITGB2-ICAM2, which are involved in costimulatory signalling and immune coordination [49,50]. In contrast, most Tn subsets, except c04, lacked such interaction (Figure S5(D and E)), suggesting limited direct involvement in B-T crosstalk.

To investigate lineage relationships, we applied STARTRAC transition index to quantify TCR sharing. In both age groups, c07 and c08 Tm subsets showed high transition propensities toward c09 Tem cells, indicating that these may serve as precursors to c09 (Figure S5(B and C)). Additionally, c04–c06 subsets showed moderate transition potential with both c07 and c09 (Figure S5(F and G)), supporting their involvement in the vaccine response.

Pseudo-time trajectory analysis revealed divergent differentiation pathways between age groups (Figure 7(F and G)). In elderly individuals, CD4+ T cells followed a relatively linear trajectory, originating from the c04_Tn, passing through c08_Tem, and ultimately differentiating into the c09_Tem. In young individuals, two differentiation trajectories were observed, including a novel branch (Figure 7(G)), characterized by progressive upregulation of TCR-related genes, such as TRDV2, TRGV9, and TRDC (Figure 7(H)). These findings suggest that the WSK-V102C vaccine more effectively activates CD4+ Tm cells in young individuals, promoting their activation, proliferation and functional diversification.

TF analysis further highlighted marked differences between age groups. KLF7, a regulator of IL-6 expression [53], was broadly upregulated in CD4+ T cell subsets from young individuals, while KLF6 was consistently downregulated (Figures 7(I) and S5(H)). Additionally, MAFB, RXRA, CEBPB, and CEBPD*,* known regulators of activation, differentiation, and inflammation, were highly expressed across multiple B cell and CD4+ T cell subsets in the young individuals (Figures 6(H), 7(I), S4(J), and S5(H)). Although these factors have established roles in aging and immune function, their specific impact on variant-directed immunity (e.g. XBB.1.5) requires further investigation. Taken together, WSK-V102C vaccination induces distinct CD4+ T cell differentiation patterns in young and elderly individuals, with younger recipients exhibiting more dynamic trajectories, stronger B–T cell interactions, and a unique transcriptional profile marked by KLF7 and inflammatory TFs. These features may contribute to more effective T cell–mediated immunity in the young.

## Discussion

In this study, we integrated single-cell transcriptomics with immune repertoire profiling to rigorously define vaccine-correlated clones and to dissect post-booster immune responses to WSK-V102C, an XBB.1.5 trivalent recombinant protein vaccine. By tracing plasmablast clonal origins and constructing a comprehensive single-cell immune atlas across age groups, we identified age-associated differences in both humoral and cellular immune organization after booster vaccination. These findings provide a descriptive view of how immune coordination differs between younger and elderly individuals following vaccination with a variant-adapted SARS-CoV-2 vaccine.

Although plasmablast responses typically peak within 7–14 days following vaccination, the present study focused on immune features at Day 28 post-booster, a time point commonly used to evaluate stabilized vaccine-induced immune responses [54,55]. This later time point reflects consolidation of germinal centre reactions and the transition from acute effector responses to antigen-specific memory compartments. Accordingly, our analyses emphasized memory B cells, circulating T follicular helper subsets, and functional cytokine programmes rather than early plasmablast dynamics. While this design does not capture transient plasmablast kinetics, it enables comparison of post-vaccination immune states during the consolidation phase.

At B cell level, we observed distinct age-associated response patterns following booster vaccination. In elderly individuals, post-vaccination B-cell features were characterized by reduced activation of naïve B-cell subsets and a greater reliance on transcriptional programmes consistent with memory-biased differentiation. In contrast, young individuals exhibited signatures indicative of broader B-cell activation, clonal expansion, and engagement of naïve B-cell compartments. Although vaccine-correlated B cells from elderly individuals exhibited higher levels of somatic hypermutation, those from young individuals displayed a proportionally greater usage of SARS-CoV-2-associated IGHV genes, suggesting differences in how prior immune memory and *de novo* activation contribute to post-vaccination responses across age groups.

Notably, neutralizing antibody titres at Day 28 correlated with the degree of vaccine-correlated B-cell clonal expansion in elderly individuals but not in younger individuals. This divergence suggests age-associated differences in the organization of humoral immune responses following booster vaccination. In elderly individuals, the humoral response appears to be predominantly driven by the quantitative recall and expansion of pre-existing memory B cell compartments, which could result in a more direct correlation between clonal expansion and antibody production. Consistent with this interpretation, clonal expansion in the elderly was characterized by the preferential expansion of a limited number of dominant B-cell clones. The combination of a memory-biased response and a more restricted clonal architecture may therefore contribute to the strong linear relationship observed between antibody titres and B-cell expansion in this age group.

In contrast, antibody responses may arise from a more complex integration of memory recall and *de novo* activation from naïve B cells in young individuals. This complexity might obscure a simple linear relationship with B cell subsets while contributing to higher overall response quality. Consistent with prior reports of age-associated alterations in germinal centre dynamics [20], these patterns may influence the breadth and durability of post-vaccination antibody response [4,21]. Notably, single-cell RNA sequencing did not reveal a marked reduction in overall B-cell abundance or subset diversity in elderly individuals [19], suggesting that booster vaccination may sustain peripheral B-cell activation despite age-associated remodelling.

Age-associated differences were also evident in CD4+ T-cell responses. At baseline, CD4+ T cells from elderly individuals exhibited transcriptional signatures consistent with prior activation and differentiation. Following booster vaccination, younger individuals displayed more prominent post-vaccination programmes associated with activation, differentiation, antigen presentation, and cytokine support for B-cell responses. Although clonal expansion was observed in both age groups, elderly individuals showed more limited shifts in TRAV/TRBV gene usage, consistent with previously reported constraints in antigen-specific T-cell repertoires in older adults [4]. These findings suggest differences in cellular immune plasticity and repertoire remodelling following vaccination.

Analysis of T-cell receptor usage further revealed that genes with higher representation in SARS-CoV-2-associated repertoires at baseline decreased in frequency after vaccination, particularly in younger individuals. This pattern suggests dynamic reshaping of TCR usage following booster immunization, whereas TCR repertoires in elderly individuals appeared more stable and memory-biased. While age-associated thymic involution and atrophy constrain T-cell receptor diversity likely contribute to these differences [15,16], alternative explanations, such as contraction of activated T-cell populations by Day 28, cannot be excluded. Given the limited availability of variant-specific TCR repertoire data, further studies will be required to validate and extend these observations.

Single-cell pseudo-time analyses further supported these age-associated patterns. In elderly individuals, B-cell trajectories showed a preferential bias toward differentiation from memory B-cell subsets into plasmablasts, whereas younger individuals exhibited bifurcated trajectories involving commitment to both plasmablast and memory B-cell lineages. Concurrently, young individuals displayed more pronounced cell-cell communication between B-cell subsets and CD4+ T cells, consistent with a higher degree of coordinated immune activation following vaccination. Together, these observations suggest that post-vaccination B-cell responses in elderly individuals are more strongly shaped by engagement of pre-existing memory compartments, with comparatively limited recruitment of naïve B cells. This response pattern is consistent with effective antibody induction through a more focused and memory-dependent pathway, in contrast to the broader and more diversified B-cell activation landscape observed in young individuals.

Transcription factor analysis revealed age-associated regulatory programmes. Coordinated enrichment of transcription factors such as CEBPB, CEBPD and MAFB was observed in both B cells and CD4+ T cells from young individuals, whereas such concordance was less evident in elderly individuals. Differential expression of KLF7 and KLF6 was also observed following booster vaccination, suggesting age-associated differences in transcriptional programmes linked to cytokine-related signalling pathways. In older adults, reduced levels of proinflammatory cytokines, particularly IL-6, have been reported to be associated with limited T-cell proliferation and activation, impaired differentiation of B cells into plasma cells, and decreased antibody production [56,57]. KLF6 is known to activate multiple genes that negatively regulate the NF-κB pathway, thereby constraining NF-κB signalling [58], whereas activation of the NF-κB pathway promotes subsequent induction of IL-6 [59]. In contrast, KLF7 has been reported to function as a transcription factor that upregulates IL-6 [53]. Notably, this divergence was observed specifically in the context of booster vaccination, in which young individuals exhibited upregulation of KLF7 together with downregulation of KLF6, whereas elderly individuals showed no significant alteration in either transcription factor. Collectively, these observations suggest that a booster-dependent, age-associated shift in the balance between KLF7 and KLF6 may linked to differences in IL-6-related transcriptional programmes, potentially contributing to distinct immune coordination patterns across age groups. The potential roles of KLF7 and KLF6 identified here are based on correlative evidence, highlighting the need for targeted functional experiments to test their direct involvement in regulating vaccine-induced immune responses across age groups.

This study has several limitations. First, as an observational, within-subject longitudinal study without an unvaccinated control group, our design identifies associations but does not permit causal inference regarding vaccine effects. Second, immune profiling was limited to a single post-booster time point, precluding assessment of early effector dynamics, long-term immune memory formation and durability. Third, the cohort was recruited from a single clinical centre and exhibited limited population diversity, which may restrict the generalizability of the findings to other geographic or ethnic populations. In addition, information on undiagnosed or unreported comorbidities, concomitant medications, and body mass index was not systematically collected or comprehensively verified. Although participants were generally healthy adults without known immunodeficiency, unmeasured health characteristics may have influenced immune responses. Finally, given the observational nature of this work, our conclusions are primarily based on transcriptomic and clonal analyses rather than direct functional assays. Accordingly, the findings should be interpreted as descriptive and hypothesis-generating, and the proposed mechanisms will require future functional validation.

In summary, this study provides a comprehensive single-cell characterization of post-vaccination immune organization following XBB.1.5-adapted booster vaccination in younger and elderly individuals. By delineating age-associated differences in B-cell and CD4+ T-cell transcriptional programmes and clonal features, our work establishes a framework for future longitudinal and functional studies aimed at understanding immune responses to variant-adapted SARS-CoV-2 vaccines across the lifespan.

## Author contributions

Chao Wu, Yuxin Chen, Shixing Chen, Jing Chen, Shengxia Yin and Fan Yang conceived the study. Shixing Chen, Jinqiu Ran, Wen Zhang, Wanying Zhang and Juan Zhang collected the clinical data for the study. Shixing Chen, Yuxin Chen and Fan Yang established and optimized the experimental protocols. Shixing Chen, Tao Liu, Fan Yang, Chen Li, Xun Wang and Pengfei Wang verified the underlying data and performed data analysis. Shixing Chen, Tao Liu, Yuxin Chen and Chao Wu prepared the first draft of the manuscript. All authors were responsible for data interpretation, critical revision of the manuscript, and approval of the version submitted for publication.

## Supplementary Material

FigureS1new.png

FigureS5new.png

Graphical Abstract.png

FigureS2new.png

FigureS4new.png

FigureS3new.png

Tables_clean.docx

## Data Availability

The raw sequencing data generated in this study have been deposited in the Genome Sequence Archive in National Genomics Data Center, China National Center for Bioinformation / Beijing Institute of Genomics, Chinese Academy of Sciences (GSA-Human: HRA011668) that are publicly accessible at https://ngdc.cncb.ac.cn/gsa-human [60,61]. The single-cell RNA seq data for the healthy control cohorts were obtained from the Gene Experssion Omnibus (GEO) under assession numbers GSE157007 and GSE213516. Further information and requests for resources should be directed to the lead contact, Yuxin Chen (yuxin.chen@nju.edu.cn).
