# Supplementary figures and images for: Age-associated differences in XBB.1.5 trivalent booster vaccine-induced adaptive responses revealed by single-cell RNA sequencing
Source: Emerg Microbes Infect. 2026 Feb 3;15(1):2627067. doi: 10.1080/22221751.2026.2627067 (PMC12912223; doi:10.1080/22221751.2026.2627067)

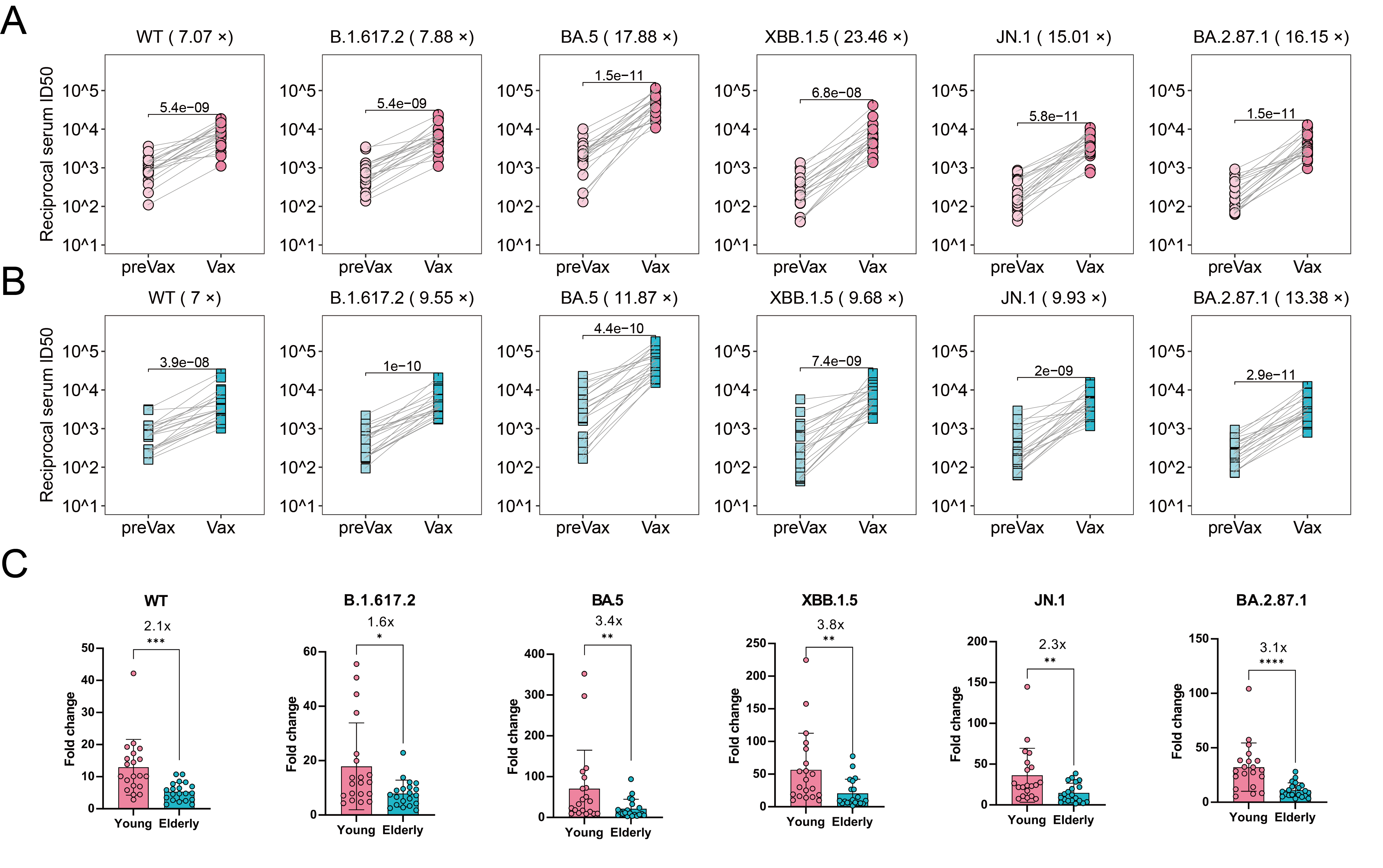

Supplement: FigureS1new.png [file TEMI_A_2627067_SM2383.png]

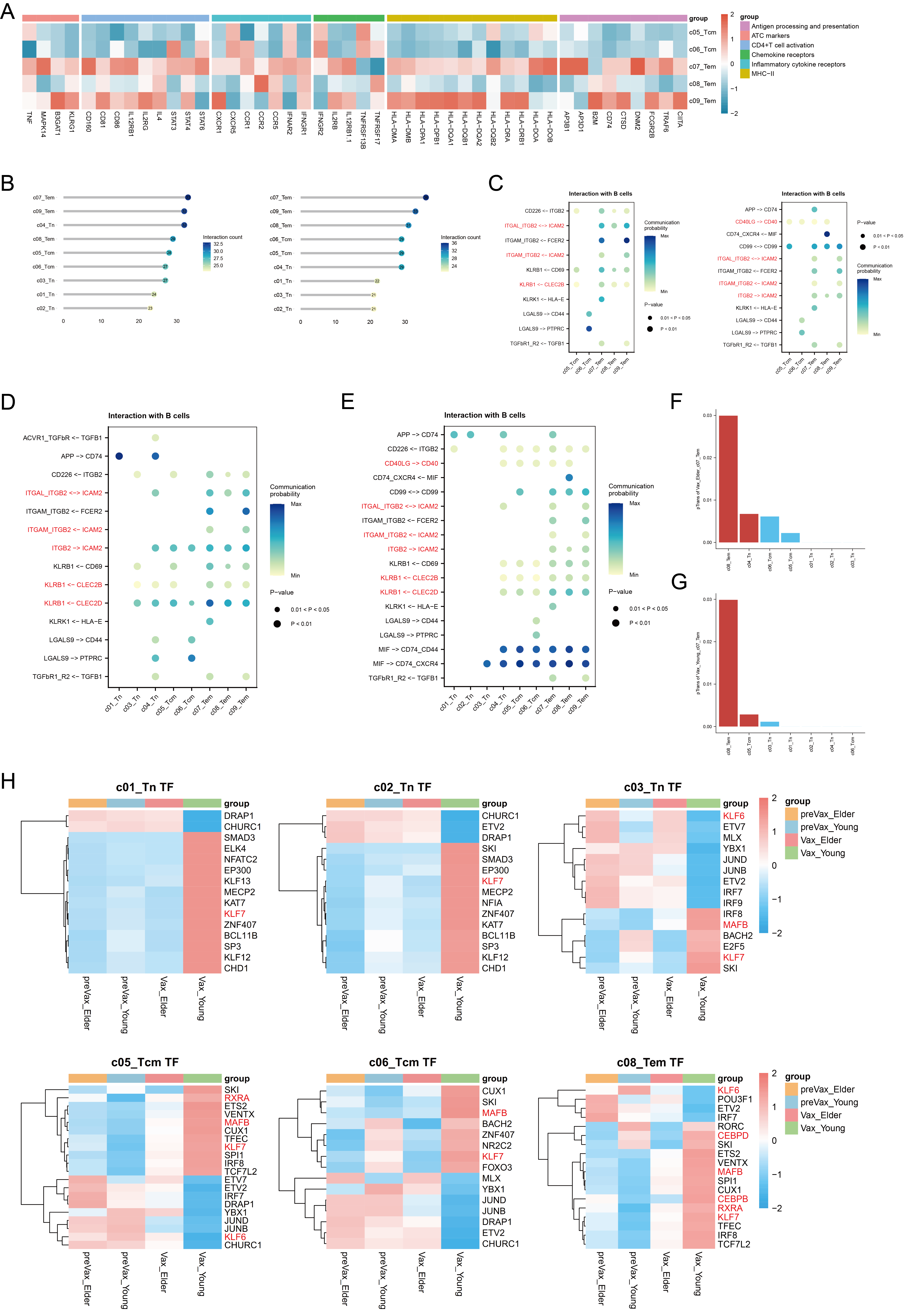

Supplement: FigureS5new.png [file TEMI_A_2627067_SM2382.png]

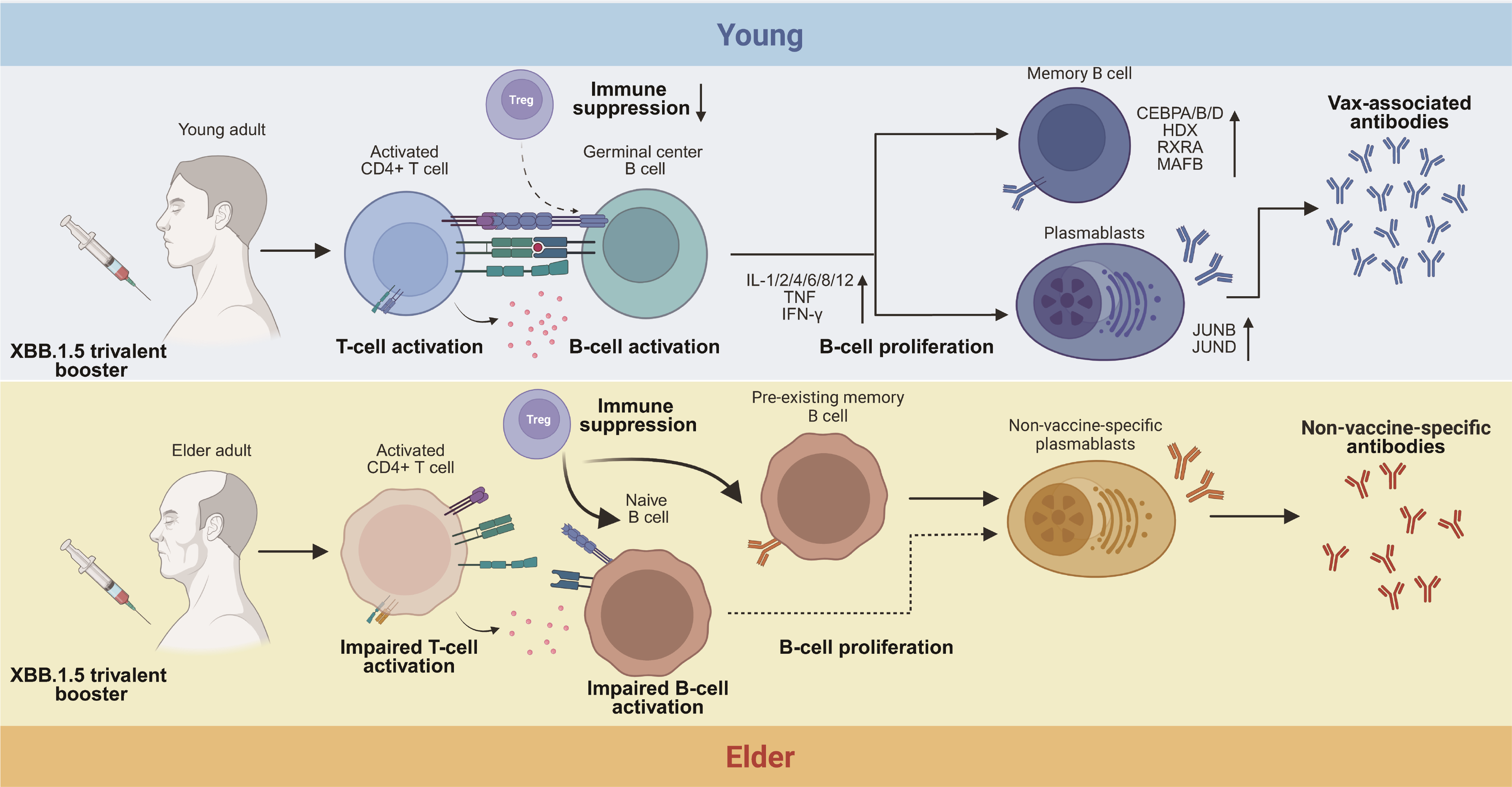

Supplement: Graphical Abstract.png [file TEMI_A_2627067_SM2381.png]

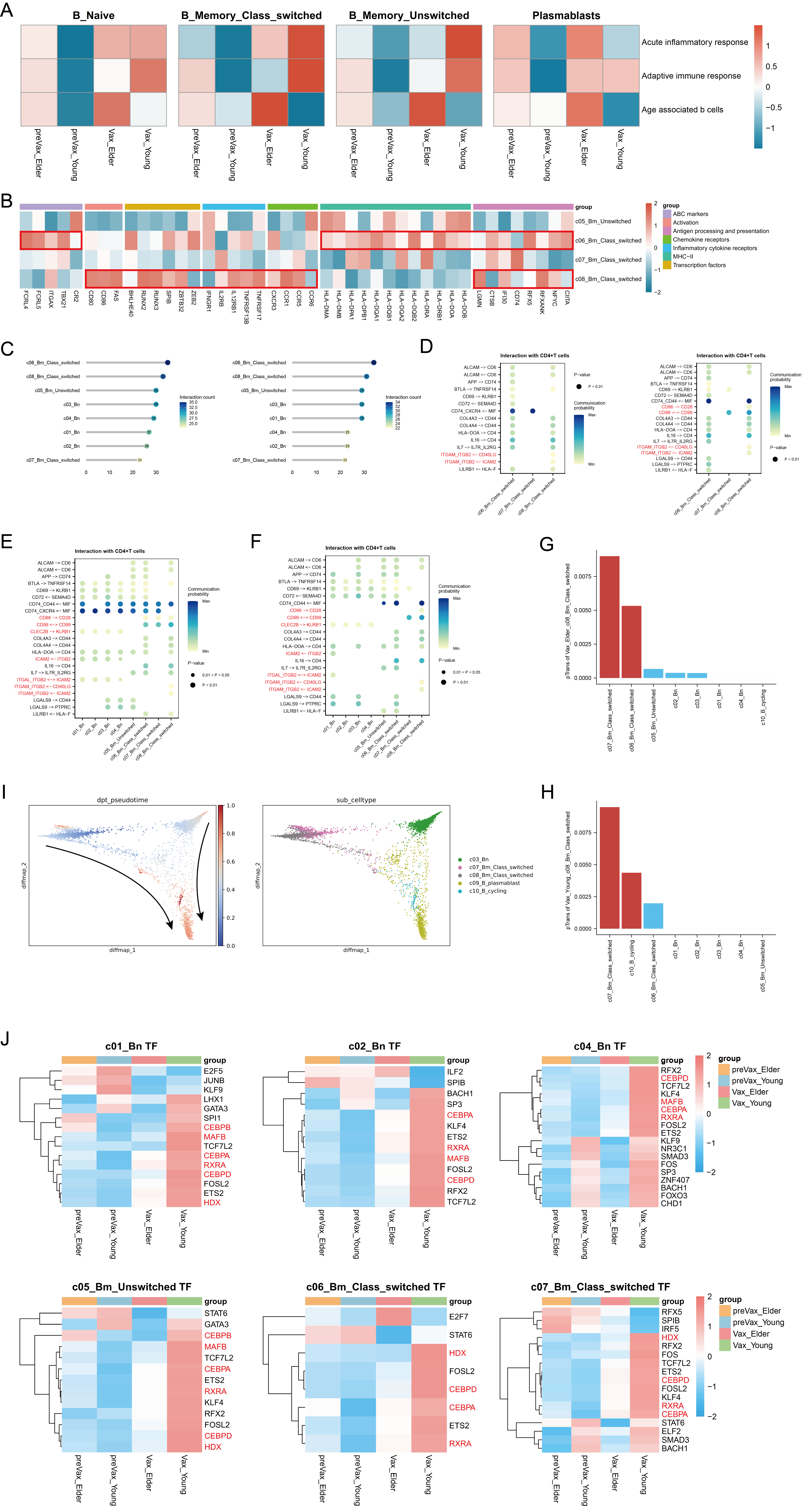

Supplement: FigureS4new.png [file TEMI_A_2627067_SM2379.png]
