# Supplementary material for: Age-associated differences in XBB.1.5 trivalent booster vaccine-induced adaptive responses revealed by single-cell RNA sequencing
Source: Emerg Microbes Infect. 2026 Feb 3;15(1):2627067. doi: 10.1080/22221751.2026.2627067 (PMC12912223; doi:10.1080/22221751.2026.2627067)
Supplement: Tables_clean.docx [file TEMI_A_2627067_SM2377.docx]

**Table S1 | Demographic and vaccination-related characteristics of elderly participants included in the scRNA-seq analysis.**

| ID | Sex | Age | Doses of COVID-19 vaccines | Number of COVID-19 Infections | Months Since Last COVID-19 Infection |
| --- | --- | --- | --- | --- | --- |
| y1 | Female | 38 | 3 | 1 | 12 |
| y2 | Male | 30 | 2 | 1 | 12 |
| y3 | Female | 25 | 2 | 2 | 12 |
| y4 | Male | 27 | 3 | 1 | 12 |
| y5 | Female | 26 | 2 | 1 | 12 |
| y6 | Female | 25 | 3 | 2 | 11 |
| y7 | Male | 35 | 3 | 1 | 12 |
| y8 | Male | 34 | 3 | 1 | 7 |
| y9 | Male | 25 | 3 | 2 | 7 |
| y10 | Male | 30 | 2 | 1 | 12 |
| y11 | Female | 28 | 3 | 1 | 20 |
| y12 | Male | 33 | 3 | 2 | 9 |
| y13 | Male | 27 | 3 | 1 | 12 |
| y14 | Male | 36 | 3 | 2 | 19 |
| y15 | Female | 34 | 2 | 1 | 12 |
| y16 | Male | 36 | 3 | 1 | 11 |
| y17 | Female | 33 | 2 | 1 | 11 |
| y18 | Female | 23 | 2 | 2 | 6 |
| y19 | Female | 26 | 3 | 1 | 12 |
| y20 | Male | 25 | 2 | 1 | 11 |
| y21 | Male | 28 | 2 | 2 | 12 |
| y22 | Female | 23 | 3 | 1 | 12 |

**Table S2 | Demographic and vaccination-related characteristics of elderly participants included in the scRNA-seq analysis**

| ID | Sex | Age | Doses of COVID-19 vaccines | Number of COVID-19 Infections | Months Since Last COVID-19 Infection |
| --- | --- | --- | --- | --- | --- |
| e1 | Female | 79 | 3 | 1 | 12 |
| e2 | Male | 78 | 3 | 1 | 12 |
| e3 | Female | 77 | 3 | 1 | 12 |
| e4 | Male | 76 | 3 | 1 | 12 |
| e5 | Female | 76 | 3 | 1 | 12 |
| e6 | Male | 76 | 3 | 1 | 12 |
| e7 | Male | 78 | 3 | 1 | 12 |
| e8 | Female | 77 | 3 | 1 | 12 |
| e9 | Female | 76 | 3 | 1 | 12 |
| e10 | Female | 86 | 2 | 1 | 12 |
| e11 | Male | 83 | 3 | 1 | 11 |
| e12 | Male | 75 | 3 | 1 | 12 |
| e13 | Male | 73 | 3 | 1 | 12 |
| e14 | Female | 73 | 3 | 1 | 12 |
| e15 | Male | 81 | 3 | 1 | 11 |
| e16 | Male | 75 | 3 | 1 | 11 |
| e17 | Female | 83 | 2 | 1 | 12 |
| e18 | Female | 74 | 3 | 1 | 12 |
| e19 | Female | 74 | 3 | 1 | 10 |
| e20 | Female | 74 | 3 | 2 | 8 |

**Table S3 | Demographic characteristics and data sources of age-matched healthy control individuals for external comparison in the scRNA-seq analysis.**

| Group | Sample ID | Age | Sex | Access Number |
| --- | --- | --- | --- | --- |
| Elderly | F020 | 78 | Male | GSE157007 |
| Elderly | F021 | 72 | Male | GSE157007 |
| Elderly | F023 | 80 | Female | GSE157007 |
| Elderly | OH14 | 88 | Female | GSE157007 |
| Elderly | F77 | 77 | Female | GSE213516 |
| Elderly | M74 | 74 | Male | GSE213516 |
| Young | F012 | 27 | Male | GSE157007 |
| Young | F014 | 23 | Female | GSE157007 |
| Young | F30 | 30 | Female | GSE213516 |
| Young | F36 | 36 | Female | GSE213516 |
| Young | M28 | 28 | Male | GSE213516 |
| Young | M36 | 36 | Male | GSE213516 |
